# Experience-Dependent Changes in Myelin Basic Protein Expression in Adult Visual and Somatosensory Cortex

**DOI:** 10.3389/fncel.2020.00056

**Published:** 2020-03-17

**Authors:** Kathryn M. Murphy, Steven J. Mancini, Katherine V. Clayworth, Keon Arbabi, Simon Beshara

**Affiliations:** ^1^McMaster Integrative Neuroscience Discovery and Study (MiNDS) Program, McMaster University, Hamilton, ON, Canada; ^2^Department of Psychology, Neuroscience & Behaviour, Faculty of Science, McMaster University, Hamilton, ON, Canada; ^3^Division of Neurology, Department of Medicine, Queen's University, Kingston, ON, Canada

**Keywords:** visual cortex (V1), somatosensory cortex, monocular deprivation, environmental enrichment (EE), myelin basic protein (MBP), myelin, adult plasticity

## Abstract

An experience-driven increase in oligodendrocytes and myelin in the somatosensory cortex (S1) has emerged as a new marker of adult cortical plasticity. That finding contrasts with the view that myelin is a structural brake on plasticity, and that contributes to ending the critical period (CP) in the visual cortex (V1). Despite the evidence that myelin-derived signaling acts to end CP in V1, there is no information about myelin changes during adult plasticity in V1. To address this, we quantified the effect of three manipulations that drive adult plasticity (monocular deprivation (MD), fluoxetine treatment or the combination of MD and fluoxetine) on the expression of myelin basic protein (MBP) in adult rat V1. In tandem, we validated that environmental enrichment (EE) increased cortical myelin by measuring MBP in adult S1. For comparison with the MBP measurements, three plasticity markers were also quantified, the spine markers drebrin E and drebrin A, and a plasticity maintenance marker Ube3A. First, we confirmed that EE increased MBP in S1. Next, that expression of the plasticity markers was affected in S1 by EE and in V1 by the visual manipulations. Finally, we found that after adult MD, MBP increased in the non-deprived V1 hemisphere, but it decreased in the deprived hemisphere, and those changes were not influenced by fluoxetine. Together, the findings suggest that modulation of myelin expression in adult V1 may reflect the levels of visually driven activity rather than synaptic plasticity caused by adult plasticity.

## Introduction

In the visual cortex (V1), the developmental increase and signaling of intra-cortical myelin are described as a structural brake on critical period (CP) plasticity (Bavelier et al., 2010). Recent studies of adult somatosensory cortex (S1), however, have shown that enhancing plasticity with environmental enrichment (EE) increases cortical oligodendrocytes and myelination (Hill et al., 2018; Hughes et al., 2018). Those increases suggest that more myelin may be a marker of adult plasticity in S1. There is no similar information about plasticity-related myelin changes in adult V1, and that gap leaves unanswered if myelin plasticity in the adult cortex might differ between S1 and V1. Here, we addressed if manipulations that are known to affect plasticity in adult rodent V1 (e.g., monocular deprivation (MD) and fluoxetine administration) cause changes to myelin expression.

The idea that myelin is a brake on CP plasticity in V1 comes from two lines of evidence. First, V1 has little expression of myelin genes (Lyckman et al., 2008) or proteins (Bjelke and Seiger, 1989; Siu et al., 2015) during the CP when abnormal visual experience (e.g., MD) quickly changes cortical function. Second, myelin signaling in V1 inhibits experience-dependent neurite growth (Schoop et al., 1997) by various myelin-associated inhibitors, including Nogo, MAG, and OMgp (Wang et al., 2002; McGee et al., 2005; Akbik et al., 2012). Knocking out the receptor for Nogo (Nogo-66, NgR) prolongs ocular dominance plasticity in V1 (McGee et al., 2005) while in the somatosensory cortex (S1) it prolongs juvenile-like dendritic spine turnover (Akbik et al., 2013).

*In vivo* imaging of oligodendrocytes in mouse S1, however, has found that myelination develops slowly past the end of the CP when myelinating cells continue to proliferate and form discontinuous patches of myelin along cortical axons in young adult animals (Hill et al., 2018; Hughes et al., 2018). Also, sensory enrichment is known to enhance plasticity in rodents (Rosenzweig and Bennett, 1996) causes a 5-fold increase in the number of oligodendrocytes and hundreds of new myelin sheaths that remain stable for at least 3 months (Hughes et al., 2018). This rapid experience-dependent increase in myelin suggests that myelination supports plasticity in the adult cortex. Furthermore, several features of myelin sheaths on cortical inhibitory neurons suggest distinct functions related to plasticity (Micheva et al., 2018). Thus, cortical myelin has a broad spectrum of functions linked to plasticity in adult S1. Less is known, however, about myelin plasticity in adult V1.

V1 of adult rodents maintains some ocular dominance plasticity, but it is less than during the CP and requires longer MD to promote a shift (Sawtell et al., 2003; Sato and Stryker, 2008). Combining MD with treatment using fluoxetine, however, reinstates juvenile-like plasticity in adult V1 (Maya Vetencourt et al., 2008) and changes the expression of many plasticity-related proteins (Beshara et al., 2015). Despite this understanding of plasticity in adult V1, there is little information about whether MD or fluoxetine in adult animals affects cortical myelin. In particular, does fluoxetine-enhanced plasticity increase myelin in V1 similar to EE driven changes in S1. To address this, we studied the expression of myelin basic protein (MBP) in adult V1 after manipulating visual experience (MD) or enhancing plasticity (fluoxetine) and compared it with MBP changes in S1 after exposure to an enriched environment (EE). Because both MD and EE affect dendritic spines (Oray et al., 2004; Jung and Herms, 2014) we also measured a pair of markers that regulate spine plasticity and synapse formation (embryonic-type drebrin E and adult-type drebrin A; Koganezawa et al., 2017; Hanamura et al., 2018). Also, an E3 ubiquitin-protein, Ube3A, was measured because it is necessary for MD driven plasticity (Yashiro et al., 2009; Sato and Stryker, 2010), long-term potentiation (Jiang et al., 1998) and there is reduced cortical expression of MBP and other myelin proteins in Ube3A deficient mice (Grier et al., 2015). We found that EE increased MBP in S1 while MD caused hemisphere-specific changes in V1, increasing MBP in the non-deprived hemisphere and decreasing it in the deprived hemisphere. Fluoxetine did not affect the experience-driven changes in MBP even though it did affect the expression of the plasticity markers (drebrin-E, drebrin-A, and Ube3A). These results suggest that the direction of myelin plasticity in the adult cortex depends on whether the neural activity is enhanced or reduced.

## Materials and Methods

### Rearing Conditions and Surgical Procedures

All procedures were approved by the McMaster University Animal Research Ethics Board. We quantified the expression of MBP and markers for dendritic spines (drebrin E and drebrin A) and plasticity maintenance (Ube3A) in S1 and V1 of adult Long-Evans rats. S1 was studied from animals reared individually (*n* = 12) or group-housed in an enriched environment (EE) consisting of multiple forms of physical stimulation including a large, spacious cage, with four levels connected by three ramps, and a running wheel (Short-term EE (S-EE) 2 weeks *n* = 6; Long-term EE (L-EE) 66 weeks *n* = 5; Critter Nation Double Unit Model:162, MidWest Homes For Pets). The toys and location of food and water were changed weekly. The lengths of EE and rearing conditions were selected to be similar to previous studies (Baroncelli et al., 2012). V1 was studied from animals reared individually with normal binocular vision (BV *n* = 6), or one of three manipulations: 1 month of fluoxetine (P70-P98, *n* = 8), 1 week of MD (P91–98; *n* = 6), 1-month fluoxetine (P70–98) plus 1 week MD (P91–98; *n* = 8). Fluoxetine was dissolved in the drinking water (0.2 mg/ml of drinking water), and animals were permitted to self-regulate their intake of food and water. These rearing conditions, including the use of male rats, were selected to be similar to previous studies (Maya Vetencourt et al., 2008).

**Figure 1:**
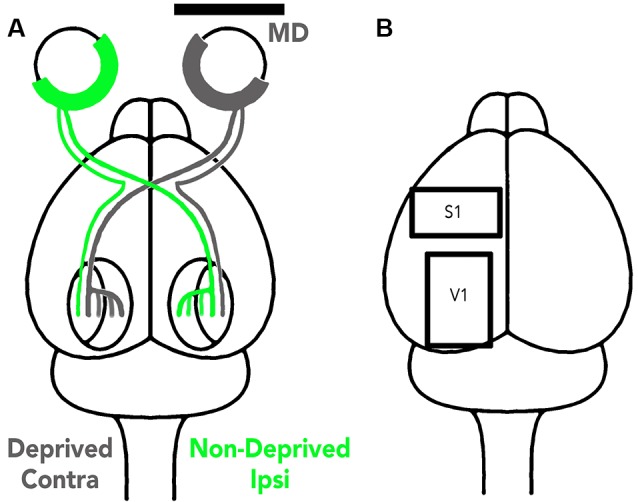
Illustration of the visual pathway to the deprived and non-deprived V1 hemispheres **(A)** and the tissue sampling regions **(B)**. **(A)** The visual pathway is illustrated using gray lines to represent the deprived eye pathway and green lines the non-deprived eye pathway. About 90% of the rat visual pathway projection projects to the contralateral hemisphere so V1 opposite the MDed eye is the deprived hemisphere while the other V1 (ipsilateral) is non-deprived because it still receives strong input from the open eye (green). **(B)** The cortical regions sampled (S1 and V1) are illustrated in one hemisphere and in this study the samples were taken from different animals.

MD was done by trimming the eyelid margins and suturing them together with 5–0 vicryl. The surgery was performed in an aseptic environment; anesthesia was induced and maintained with gaseous isoflurane (1.5–5%) in oxygen. Eyelids were monitored daily to check for and repair any openings. In the rat, 90% of the visual pathway is crossed so the hemisphere contralateral to the MDed eye was designated the deprived hemisphere and the hemisphere ipsilateral to the MDed eye the non-deprived hemisphere because it still received strong visual stimulation from the open eye (Figure 1A).

### Tissue Collection

Animals were euthanized with Euthanyl (sodium pentobarbital, 150 mg/kg) and perfused with cold 0.1 M phosphate-buffered saline (PBS; 4°C; 4–5 ml/min). The brain was removed from the skull, placed in cold PBS, and tissue samples were collected from S1 for the animals reared with EE and V1 for the other animals (Figure 1B). The samples were immediately frozen on dry ice and stored in a −80°C freezer.

### Sample Preparation and Immunoblotting

The samples were prepared from the cortical tissue pieces, protein concentrations were carefully equated, and immunoblotting was done using procedures that have been described previously (Beston et al., 2010; Murphy et al., 2014; Beshara et al., 2015; Balsor and Murphy, 2018). Importantly, since expression levels of housekeeping proteins such as GAPDH and β-actin can be inconsistent (Lee et al., 2016; Butler et al., 2019), we followed current best practices for Western blotting (Pillai-Kastoori et al., 2020) with a rigorous multi-step protocol using stringent quality control checks at each stage of the sample preparation and immunoblotting (Balsor and Murphy, 2018). Protein concentrations were equated using three replicates of each sample and bicinchoninic acid (BCA) assay (Pierce, Rockford, IL, USA). The colorimetric change was quantified (iMark Microplate Absorbance Reader, Bio-Rad Laboratories, Hercules, CA, USA) for the samples and standards and analyzed to ensure a correlation of >0.99 was achieved. The samples were diluted to 1 μg/μl with sample (M260 Next Gel Sample loading buffer 4×, Amresco) and Laemmli buffer (Cayman Chemical, Ann Arbor, MI, USA). A control sample was made from a small amount of each sample, run on every gel and used to normalize the bands for each sample run on a gel. Importantly, to maintain quality control at each step of the experiment, a high-quality pipette (Picus, Sartorious) was used and the calibration was checked daily. Each sample was run three or four times, and a total protein stain was used to normalize each lane.

The samples (25 μg) were separated on 4–20% Tris-Glycine gels (Novex, WedgeWell Gels, Thermo Fisher Scientific, Waltham, MA, USA), transferred to polyvinylidene difluoride (PVDF-FL) membranes (Millipore, Burlington, MA, USA), and membranes were blocked with blocking buffer (Odyssey Blocking Buffer 1:1 with PBS, 1 h; LI-COR Biosciences, Lincoln, NE, USA). Membranes were incubated in primary antibody overnight at 4°C (MBP, 1:4,000 Abcam; Ube3A, 1:1,000 Bethyl Laboratories, Montgomery, TX, USA; drebrin, 1:500 Fitzgerald) and PBS-T (Sigma-Adrich, St. Louis, MO, USA; 3 × 10 min). The membranes were incubated in secondary antibody (anti-mouse, 1:8,000; anti-rabbit, 1:10,000; LI-COR Biosciences, Lincoln, NE, USA) for 1 h at RT and washed in PBS. Blots were scanned (Odyssey scanner, LI-COR Biosciences, Lincoln, NE, USA) to visualize the bands, then stripped (Blot Restore Membrane Rejuvenation Kit, Millipore, Burlington, MA, USA) and reprobed with the next antibody.

### Analyses

Densitometry was used to analyze the bands (Licor Odyssey Software version 3.0; LI-COR Biosciences, Lincoln, NE, USA; Beshara et al., 2015). The control sample run on every gel was used to normalize each band on the blot and full blots are available in the Supplementary Datasheet S1.

To examine expression levels of protein expression, we plotted histograms of the mean and SEM, for each condition normalized to the control group. To compare between-the-groups we used the bootstrapping method described previously (Beshara et al., 2015). Briefly, the programming language R was used to simulate a dataset of 1,000,000 points with the same mean and SEM as the group being compared. A Monte Carlo simulation was run to compare the groups by randomly sampling *N* times from the simulated dataset where *N* was the number of animals in the comparison group (e.g., *N* = 6) and repeating this step 100,000 times to generate the expected distribution for *N* animals. Confidence intervals (CI) were calculated from the expected distribution and compared with the observed mean of the group. Groups were considered to be significantly different (i.e., *p* < 0.05) when the observed mean was outside the 95% CI of the simulated distribution. For each comparison between groups, we ran the bootstrap analysis in both directions, and the more conservative result of the significance test was reported.

Finally, we analyzed the patterns of protein expression changes in V1 for the deprived (contralateral) and non-deprived (ipsilateral) hemispheres by combining the measurements of MBP and Ube3A from this study with data for GluA2, PSD95, Gephyrin, Synapsin, and Synaptophysin from our previous study (Beshara et al., 2015). Hierarchical cluster analysis using the Ward D2 method (Murtagh and Legendre, 2014) was run in R for each condition using all seven proteins and the dendextend function in the Hmisc package. All pairwise Pearson’s R correlations were calculated using rcorr and visualized using heatmap2 in gplots. The proteins were ordered in the matrices using the dendrogram from the hierarchical clustering so proteins with similar patterns were nearby in the matrix. Each correlation in the matrix was color-coded to facilitate visualizing clusters. A table of the Pearson’s *R* values is in Supplementary Datasheet S2.

### Image Manipulation

The bands in each figure are representative of the group and full blots are in the Supplementary Datasheet S1. The size of the bands was adjusted using a uniform horizontal and vertical transformation, and a single gray-level linear adjustment was applied to all of the bands to preserve the relative intensity of the bands in the different groups.

## Results

### Effects of EE, Fluoxetine, MD and Fluoxetine Plus MD on Spine Markers

First, we examined whether the treatments had an effect on plasticity in the cortical areas using drebrin E and drebrin A as markers of dendritic spines. We chose these markers because both EE and MD are known to change spines in S1 and V1, respectively (Oray et al., 2004; Jung and Herms, 2014). Furthermore, there is a developmental shift from more of the immature protein drebrin E to more of the mature protein drebrin A, and it is that increase in drebrin A that facilitates spine maturation (Koganezawa et al., 2017).

**Figure 2:**
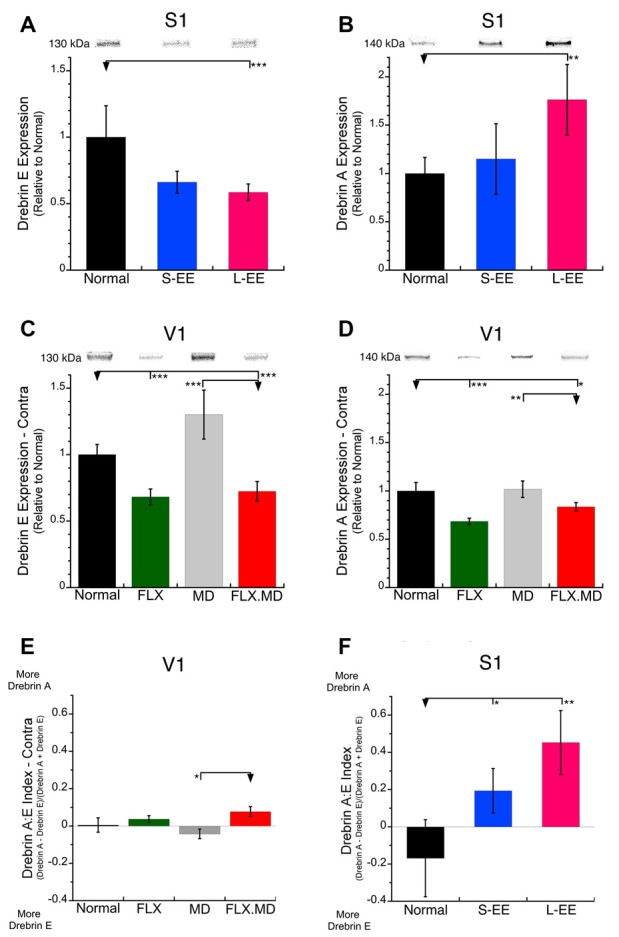
Expression of drebrin-E and drebrin-A in S1 and the deprived V1. **(A,B)** Drebrin E and drebrin A expression in S1 of control animals (*n* = 12), short-term environmental enrichment (S-EE; *n* = 6) and long-term EE (L-EE; *n* = 5). **(A)** In S1, expression of the immature drebrin E was reduced after L-EE (−41%, SEM 6.2%, *p* < 0.001). **(B)** In contrast, the mature drebrin A was increased after L-EE (+76%, SEM 36%, *p* < 0.01). **(C,D)** Drebrin E and drebrin A expression in V1 of animals reared with normal binocular vision (*n* = 6), 1-month fluoxetine (P70–98, *n* = 6), 1 week monocular deprivation (MD; P91–98, *n* = 8) or 1-month fluoxetine (P70–98) plus 1 week MD (P91–98, *n* = 8). In V1, both drebrin E and drebrin A were reduced after fluoxetine alone (FLX: drebrin-E: −32%, SEM 6.0%, *p* < 0.001; drebrin-A: −32%, SEM 3.2%, *p* < 0.001) or when fluoxetine was combined with MD (FLX MD: drebrin-E: −28%, SEM 7.4%, *p* < 0.001; drebrin-A: −17%, SEM 4.4%, *p* < 0.05). **(E,F)** An index of the relative expression of drebrin A and drebrin E was calculated where values <0 indicate more drebrin E and values >0 more drebrin A. **(E)** In V1, none of the treatment groups shifted the drebrin balance compared with normal. **(F)** In S1, both S-EE and L-EE shifted the drebrin balance to more drebrin A (S-EE *p* < 0.05, L-EE *p* < 0.01). *p < 0.05, **p < 0.01, ***p < 0.001.

In S1, the long-term EE group (−41%, SEM 6.2%, *p* < 0.001) had less drebrin E expression than normal suggesting that this treatment led to fewer nascent spines (Figure 2A). Short-term EE also appeared to have less drebrin E but that difference was not significant. In contrast, drebrin A expression was increased after long-term EE (+76%, SEM 36%, *p* < 0.01; Figure 2B), which is consistent with previous studies showing greater stability and larger mature spines after EE (Jung and Herms, 2014).

In V1, we focused on the deprived hemisphere because it is where previous studies found that MD drives spine plasticity (Oray et al., 2004). One week of MD did not significantly change the expression of either drebrin isoform compared with normals and there was only a trend toward increased expression of drebrin E (MD contra, +30%, SEM 18%, n.s.; Figures 2C,D). Fluoxetine treatment, however, whether alone or combined with MD reduced the expression of both drebrin isoforms in V1 (drebrin E: −32%, SEM 6.0%, *p* < 0.001; drebrin A: −32%, SEM 3.2%, *p* < 0.001; Figures 2C,D). This is one of the few plasticity-related synaptic markers in V1 that is affected by fluoxetine (Beshara et al., 2015).

Since the drebrin isoforms regulate different aspects of spine maturation and there is a developmental shift in the balance between the markers (immature drebrin E; mature drebrin A) we calculated an index to determine if the treatments changed the balance between the drebrin isoforms (Figures 2E,F). The control animals in both V1 and S1 had a balanced expression of drebrin A and E resulting in index values that overlapped zero. In V1, the drebrin isoforms balance was not changed by fluoxetine, MD or the combination of treatments (Figure 2E). In contrast, both short-term (*p* < 0.05) and long-term EE (*p* < 0.01) caused a shift to more drebrin A in S1 than found in the normal young adult (Figure 2F). Taken together, we found that fluoxetine and EE drove different patterns of drebrin plasticity.

### Effects of EE on MBP and Ube3A in Somatosensory Cortex

We examined if short-term or long-term EE changed MBP expression in S1. MBP makes up about 30% of all myelin proteins and is comprised of two families: classic- (18.5–21.5 kDa) and Golli-MBP (33–35 kDa). In this study, we quantified the Classic-MBP isoform because it is found in mature oligodendrocytes and myelin sheaths, and is necessary for activity-driven compaction of myelin around axons (Wake et al., 2011).

**Figure 3:**
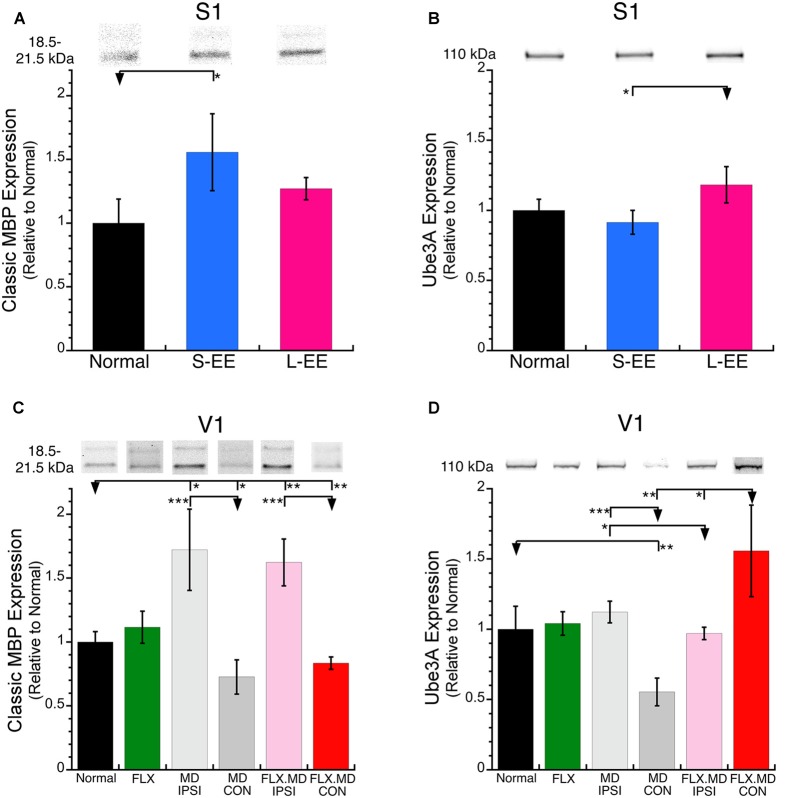
Expression of myelin basic protein (MBP) and Ube3A in S1 and V1. **(A)** S-EE increased MBP expression (+56%, SEM 30%, *p* < 0.05) and **(B)** L-EE increased Ube3A (+18%, SEM 13%, *p* < 0.05). **(C)** MBP expression was increased in the non-deprived (ipsilateral) hemisphere after both MD (light gray; MD ipsi, +72%, SEM 32%, *p* < 0.05) and fluoxetine plus MD (pink; flx MD ipsi, +62%, SEM 18%, *p* < 0.01). There was a loss of MBP expression in the deprived (contralateral) hemisphere after both MD (dark gray; MD con, −27%, SEM 13%, *p* < 0.05) and fluoxetine plus MD (red; flx MD con, −17%, SEM 4.8%, *p* < 0.01). **(D)** There was a loss of Ube3A expression after MD (MD con, −45%, SEM 9.9%, *p* < 0.001) but an increase after MD plus fluoxetine (flx MD con, +56%, SEM 33%, *p* < 0.01). *p < 0.05, **p < 0.01, ***p < 0.001.

MBP was increased after short-term EE (+56%, SEM 30%, *p* < 0.05) and appeared to be increased after long-term EE but it was not significant (+27%, SEM 9%, *p* < 0.06; Figure 3A). Those MBP results show a similar pattern to the EE driven increase reported for oligodendrocytes and myelination in S1 (Hughes et al., 2018). Next, we quantified Ube3A which is necessary for the maintenance of CP experience-dependent plasticity (Yashiro et al., 2009). Ube3A after both the short- and long-term EE was not different from normal, however, the long-term EE group had greater Ube3A expression than the short-term EE group (Figure 3B).

### Effects of Fluoxetine and MD on MBP and Ube3A in the Visual Cortex

We examined how fluoxetine, MD and the combination of treatments changed the expression of MBP and Ube3A in V1 (Figure 1B). Fluoxetine alone did not change the expression of MBP (Figure 3C) or Ube3A in V1 (Figure 3D) which contrasts with the reduced expression of both drebrin isoforms (Figures 2C,D). MD, however, caused hemisphere specific changes to MBP expression (Figure 3C). In the non-deprived (ipsi) hemisphere, there was an increase in MBP (MD ipsi: +72%, SEM 32%, *p* < 0.05) while in the deprived (contra) hemisphere there was a loss of MBP (MD con: −27%, SEM 13%, *p* < 0.05). When fluoxetine was combined with MD it did not affect the pattern of MBP expression found after MD alone and there continued to be increased expression in the non-deprived hemisphere and loss of expression in the deprived hemisphere (flx MD ipsi: +62%, SEM 18%, *p* < 0.01; flx MD con: −17%, SEM 4.8%, *p* < 0.01; Figure 3C).

The pattern of Ube3A changes in V1 did not follow MBP changes. MD caused no change in Ube3A in the non-deprived (ipsi) hemisphere but a loss in the deprived hemisphere (MD con: −45%, SEM 9.9%, *p* < 0.001; Figure 3D). The combination of fluoxetine and MD caused a recovery of Ube3A in the deprived hemisphere (flx MD con: +56%, SEM 33%, *p* < 0.01; Figure 3D). While MBP was bidirectionally changed driven by visual experience, increasing in the non-deprived and decreasing in the deprived hemisphere, Ube3A only decreased in the deprived hemisphere. Furthermore, fluoxetine treatment did not affect MBP, but it did affect the other plasticity markers (drebrin and Ube3A), especially when it was combined with MD.

**Figure 4:**
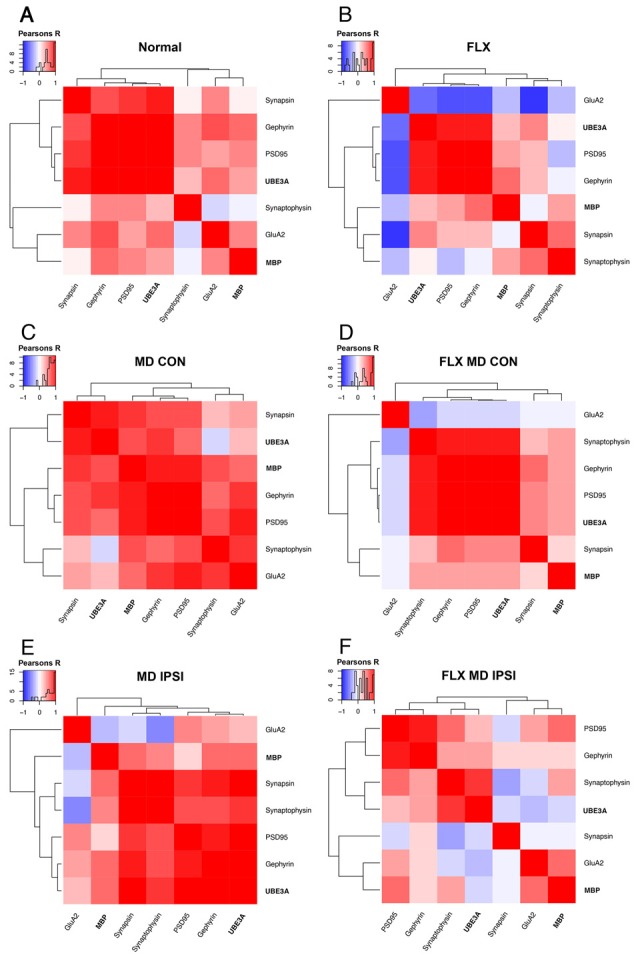
Hierarchical cluster analysis of adult V1. The high dimensional pattern of protein expression changes in V1 for the deprived (contralateral) and non-deprived (ipsilateral) hemispheres were analyzed by combining the measurements of MBP and Ube3A from this study with data for GluA2, PSD95, Gephyrin, Synapsin, and Synaptophysin from our previous study (Beshara et al., 2015). Correlation matrices are plotted to show the strength and direction (blue: negative; red: positive) of the pairwise Pearson’s R correlations between proteins for each condition and hemisphere. The inset with each panel shows the color-code and distribution of *R* values. The order of proteins was determined using unsupervised hierarchical clustering such that proteins with stronger correlations were nearby in the matrix: **(A)** normal, **(B)** fluoxetine, **(C)** MD contralateral (deprived) hemisphere, **(D)** fluoxetine and MD contralateral (deprived) hemisphere, **(E)** MD ipsilateral (non-deprived) hemisphere, and **(F)** fluoxetine and MD ipsilateral (non-deprived) hemisphere.

We used unsupervised hierarchical cluster analysis of the data from the deprived (contralateral) and non-deprived (ipsilateral) V1 hemispheres to identify high dimensional patterns in the data. Along with the expression of MBP and Ube3A, protein expression was included for five markers of synaptic plasticity (GluA2, PSD95, Gephyrin, Synapsin, and Synaptophysin) measured previously using tissue samples from the same animals (Beshara et al., 2015). MBP and Ube3A were in separate clusters for both hemispheres of all conditions (Figure 4). In normal animals, MBP clustered with GluA2 while Ube3A clustered with Synapsin, Gephyrin, and PSD95 (Figure 4A). Fluoxetine changed the relationship between MBP and GluA2 to a negative correlation and partitioned them into different clusters, but the Ube3A cluster remained similar to the normal pattern (Figure 4B). After MD, MBP clustered with the postsynaptic markers Gephyrin and PSD95 in the deprived hemisphere while Ube3A clustered with the presynaptic marker Synapsin (Figure 4C). The combination of fluoxetine with MD led to a different pattern in the deprived hemisphere. There MBP clustered with Synapsin while Ube3A clustered with the postsynaptic markers and Synaptophysin (Figure 4D).

In the non-deprived hemisphere, the pattern for MBP and GluA2 was different from normal and those proteins were partitioned into separate clusters (Figure 4E). Ube3A, however, clustered with the same synaptic markers as in normal animals (Figure 4E). Finally, the combination of fluoxetine and MD led to a unique pattern of clusters with MBP and GluA2 in a cluster and Ube3A and Synaptophysin in another cluster (Figure 4F). Interestingly, the increase in MBP in the non-deprived hemisphere led to a negative relationship with GluA2 (Figure 4E), but adding fluoxetine changed it to a positive relationship (Figure 4F) that was similar to the normal pattern between MBP and GluA2 (Figure 4A). Together, these cluster analyses showed that the combination of fluoxetine with MD changed the relationships of MBP with this collection of plasticity markers even though the expression levels of MBP were similarly affected by MD alone or with fluoxetine (Figure 3C).

## Discussion

In this study, we quantified MBP expression in S1 and V1 after treatments known to affect adult plasticity in those cortical areas. Sensory enrichment caused an increase in MBP expression in S1 that was similar to a previous finding of an increase in the number of oligodendroctyes (Hughes et al., 2018). Furthermore, the S1 changes to markers of dendritic spines (drebrin A) and plasticity maintenance (Ube3A) were consistent with previous studies showing that enrichment causes an increase in spines (Jung and Herms, 2014) and functional plasticity (Polley et al., 2004). The changes in V1 after MD and fluoxetine treatments were more complicated than the EE driven changes in S1. The increase in the nascent spine marker drebrin E in the deprived hemisphere was consistent with an MD-driven increase in spine motility (Oray et al., 2004; Hofer et al., 2008). The MBP levels in V1, however, appeared to reflect activity levels with increased MBP in the non-deprived hemisphere and decreased MBP in the deprived hemisphere. The high dimensional cluster analyses uncovered more subtle changes including showing that fluoxetine did affect the overall pattern of relationships between MBP and the other proteins. Together, these findings suggest that multiple mechanisms, including activity levels and specific plasticity mechanisms, may be involved in regulating experience-dependent changes in MBP expression in adult V1.

Much of existing research on the role of myelin in V1 plasticity has focused on the CP when myelin is typically viewed as a structural brake on visual plasticity (Bavelier et al., 2010). Thus, it was unexpected to find that MD in adults changed MBP. In the deprived hemisphere, the loss of MBP led to clustering with PSD95 and Gephyrin, which are also reduced by MD in adults (Beshara et al., 2015). In the non-deprived hemisphere, MBP increased, but the other markers are not changed (Beshara et al., 2015). That increase in MBP partitioned it into an individual cluster, suggesting that it had weak or no relationships with the other plasticity markers. Glutamatergic receptor proteins, however, are known to go through transient changes during MD and can return to normal levels within a week (Williams et al., 2015). Thus, the MBP changes measured here may reflect the longer-term impact of fluctuations in neural activity that can support more efficient neural transmission (Fields, 2015). It is well-known that neuronal activity can increase myelination (Demerens et al., 1996; Gibson et al., 2014) and perhaps the increased MBP contributes to increased metabolic support (Saab et al., 2013; Philips and Rothstein, 2017) for more active neurons in the non-deprived V1. In that framework, myelin in adult V1 could serve an adaptive role supporting responses to heightened demands of the increased visually driven activity. New studies will be needed to tease apart the mechanisms that contribute to regulating these activity-dependent MBP changes in adult V1 to determine the role of indirect signaling vs. direct glutamatergic synaptic input to oligodendrocyte precursors (OP). For example, OP cells express AMPA receptors and signaling through those receptors can stimulate the production of white matter myelin during development (Kougioumtzidou et al., 2017).

In young adult rats, 1 month of fluoxetine treatment reinstates juvenile-like ocular dominance plasticity (Maya Vetencourt et al., 2008) and increases spine density and size (Ampuero et al., 2010). Here, we did not find an effect of fluoxetine on the amount of MBP expression in V1. There was no change in MBP levels after 1 month of fluoxetine and no recovery of the MD-induced loss of MBP when fluoxetine was combined with MD. In contrast, there was evidence from drebrin and Ube3A that fluoxetine alone or combined with MD affected V1 because the drebrin isoforms were reduced, and Ube3A was increased in the deprived hemisphere. Furthermore, the cluster analysis showed that the pattern of relationships between MBP expression and plasticity markers measure in a previous study (Beshara et al., 2015) was changed by fluoxetine. Fluoxetine is known to regulate NMDA and AMPA receptors (Szasz et al., 2007; Kiss et al., 2012; Vizi et al., 2013; Barygin et al., 2017) and both receptors are found on oligodendroglia where they participate in myelination (Káradóttir and Attwell, 2007; Kougioumtzidou et al., 2017). Thus, fluoxetine could have a direct effect on the regulation of MBP in adult V1 separate from the effects on synaptic plasticity mechanisms.

Ube3A deficient mice have an experience-dependent loss of dendritic spines in V1 (Kim et al., 2016), increased excitability, weaker orientation tuning (Wallace et al., 2017) and a 25% reduction in cortical MBP (Grier et al., 2015). Here, the Ube3A and MBP changes caused by MD and fluoxetine were not the same. MD reduced both Ube3A and MBP by similar amounts in the deprived hemisphere, but only MBP was increased in the non-deprived hemisphere. Although adding fluoxetine rescued the MD-induced loss of Ube3A, it did not rescue MBP expression. It is interesting to note that all of the high dimensional analyses partitioned Ube3A and MBP into different clusters suggesting that MBP and Ube3A reflect different aspects of adult V1 function and plasticity.

In S1, the relationships between Ube3A, drebrin, and MBP after EE found here were more consistent with previous results about the effects of EE on the somatosensory cortex. For example, long-term EE increased both Ube3A and drebrin A with a trend to increased MBP, providing additional support for the idea that EE maintains enhanced adult plasticity by increasing spines (Jung and Herms, 2014) and myelin (Hughes et al., 2018). The V1 findings suggest a more complicated relationship and raise new questions about how MBP and the various components of myelin interact with other mechanisms to enhance or reduce plasticity in the adult cortex.

The experience-dependent changes in MBP expression in the adult cortex probably involve many cortical circuits, including a subset of GABAergic neurons: the fast-spiking parvalbumin-positive (PV+) cells (Kawaguchi and Kubota, 1997) that play an essential role in stimulus-selective response potentiation in adult V1 (Kaplan et al., 2016). In both mice and the human cortex, the axons of many PV+ neurons have patches of myelination that are mainly confined to the proximal axonal segment (Micheva et al., 2016; Stedehouder et al., 2017). Furthermore, the myelin sheaths around PV+ axons have more MBP than non-GABAergic axons (Micheva et al., 2018). Neuronal activity in S1 elongates the myelin patches on PV+ interneurons (Stedehouder et al., 2018), raising the possibility that increased MBP in the non-deprived V1 may reflect changes in PV+ cells myelination. In contrast, PV+ cells do not contribute to MD-driven ocular dominance in the deprived adult V1 (Kaplan et al., 2016), so the loss of MBP found there may involve other cell types. It will be necessary to follow-up these findings with high-resolution anatomical studies to determine the cells and circuits in V1 where MD changes MBP.

## Conclusions

The current findings and those of other recent studies highlight that increases in cortical myelin can be more than a structural brake on experience-dependent plasticity. Instead, cortical myelin may have a role in adaptive plasticity in the adult cortex. Future studies are needed to address the functional contributions that experience-dependent changes in cortical myelin have on adult plasticity. Those studies will be timely since interventions, such as the use of the antihistamine clemastine, are being tested to increase cortical myelin in both neurodegeneration and neurodevelopmental disorders (Liu et al., 2016; Green et al., 2017; Barak et al., 2019).

## Data Availability Statement

The Western Blot data used to support the findings of this study are available from the corresponding author upon request.

## Ethics Statement

The animal study was reviewed and approved by McMaster University Animal Research Ethics Board.

## Author Contributions

KM designed the research, analyzed the data, and wrote/revised the article. SM designed the research, performed research, analyzed the data, and wrote/revised the article. KC performed research and analyzed the data. KA analyzed the data and revised the article. SB designed the research, performed research, analyzed the data, and wrote/revised the article.

## Conflict of Interest

The authors declare that the research was conducted in the absence of any commercial or financial relationships that could be construed as a potential conflict of interest.
