# Supplementary material for: Experience-Dependent Changes in Myelin Basic Protein Expression in Adult Visual and Somatosensory Cortex
Source: Front Cell Neurosci. 2020 Mar 17;14:56. doi: 10.3389/fncel.2020.00056 (PMC7098538; doi:10.3389/fncel.2020.00056)

Figure 2 Blot to band key

Fig 2A S1 Drebrin E (130 kDa)
- Normal, blot B, lane 7
- S-EE, blot D, lane 4
- L-EE, blot D, lane 7

Fig 2B S1 Drebrin A (140 kDa)
- Normal, blot C, lane 8
- S-EE, blot B, lane 5
- L-EE, blot C, lane 3

Fig 2A&B Blot B

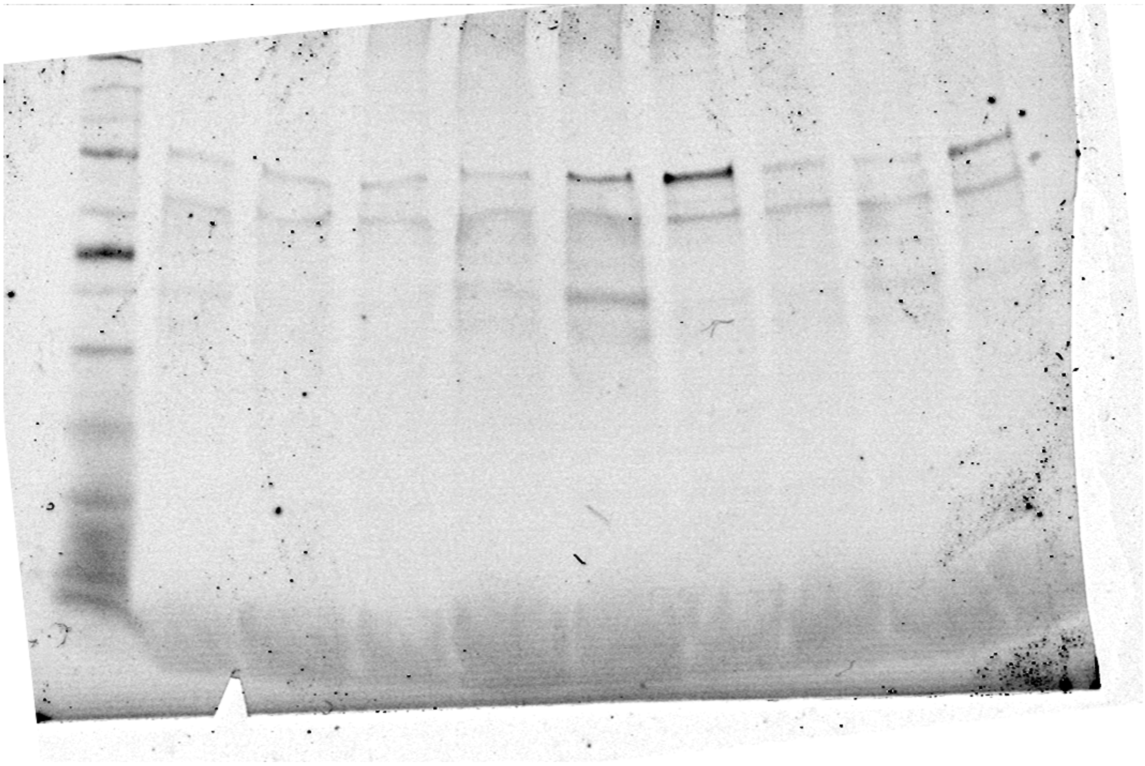

Fig 2A&B Blot C

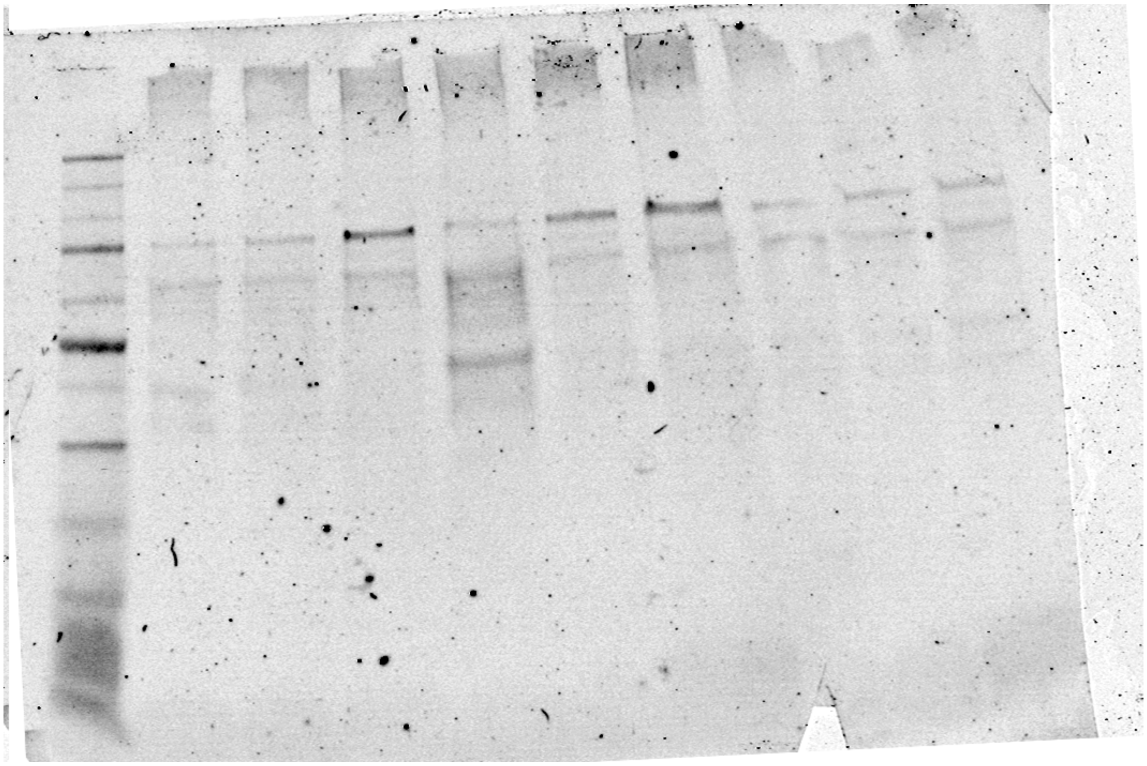

Fig 2A&B Blot D

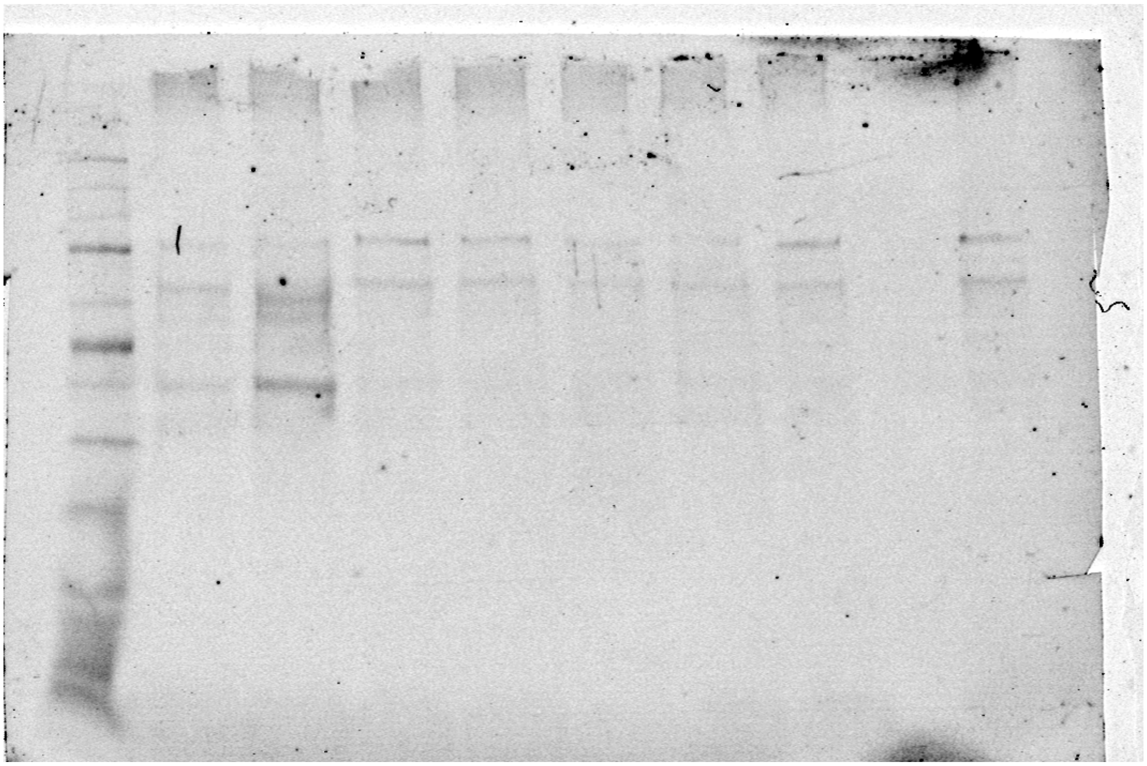

Fig 2C V1 Drebrin E (130 kDa)
- Normal, blot C, lane 6
- Fix, blot A, lane 4
- MD, blot D, lane 7
- Fix.MD, blot B, lane 4

Fig 2D V1 Drebrin A (140 kDa)
- Normal, blot C, lane 6
- Fix, blot A, lane 8
- MD, blot D, lane 7
- Fix.MD, blot B, lane 3

Fig 2 C&D Blot A

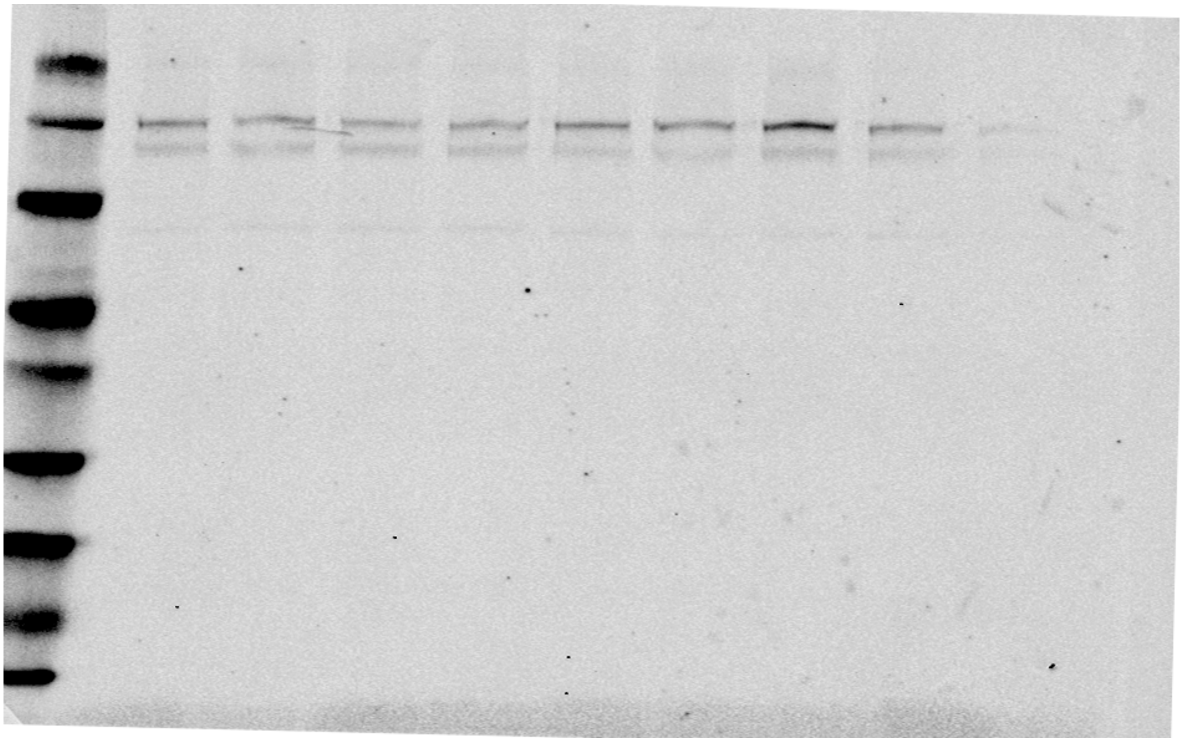

Fig 2 C&D Blot B

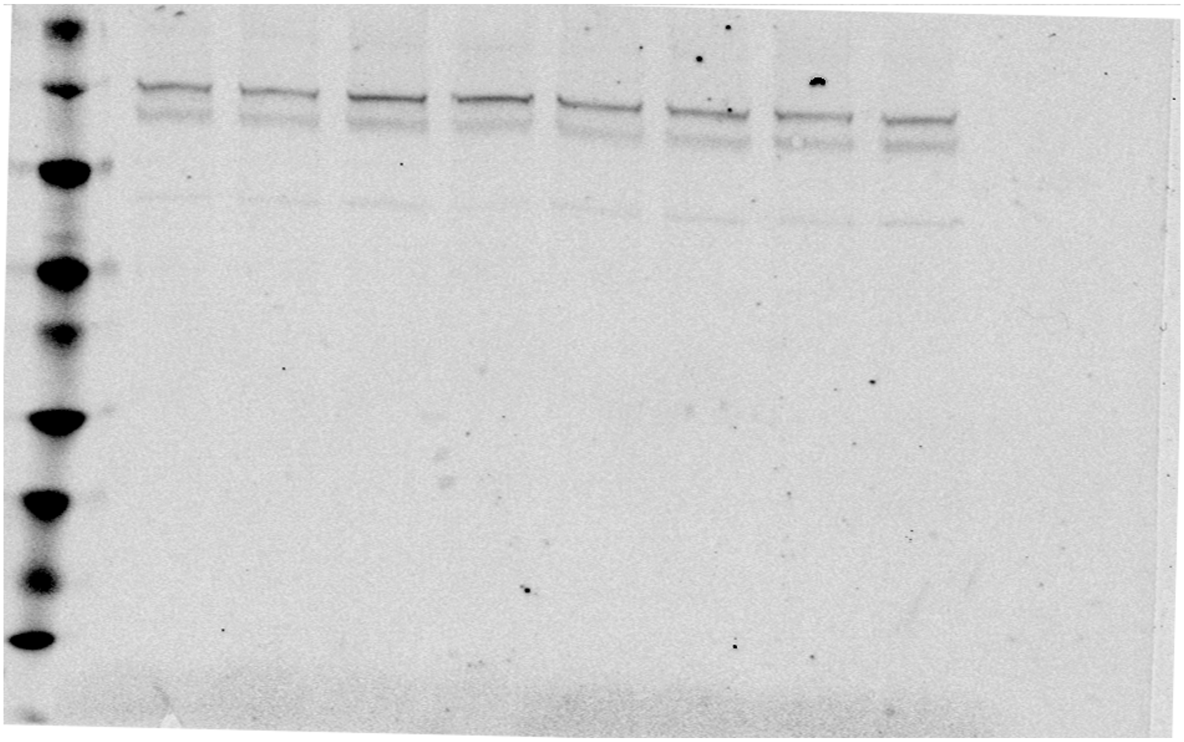

Fig 2 C&D Blot C

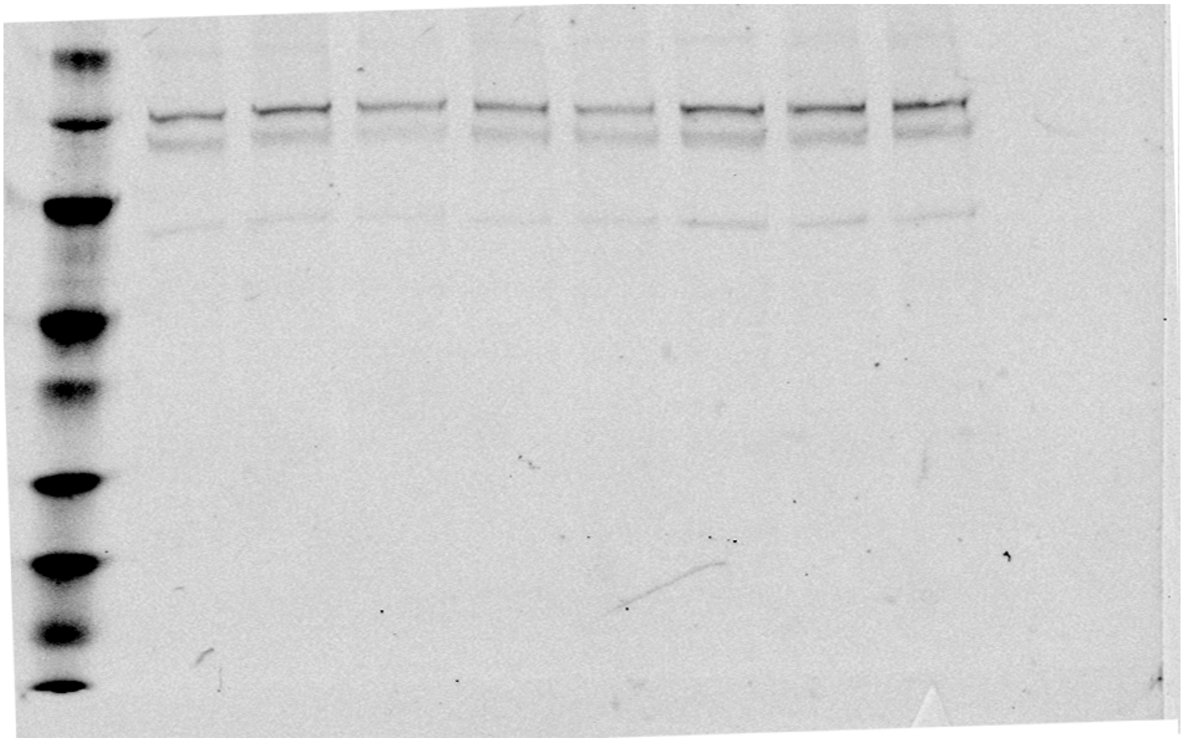

Fig 2 C&D Blot D

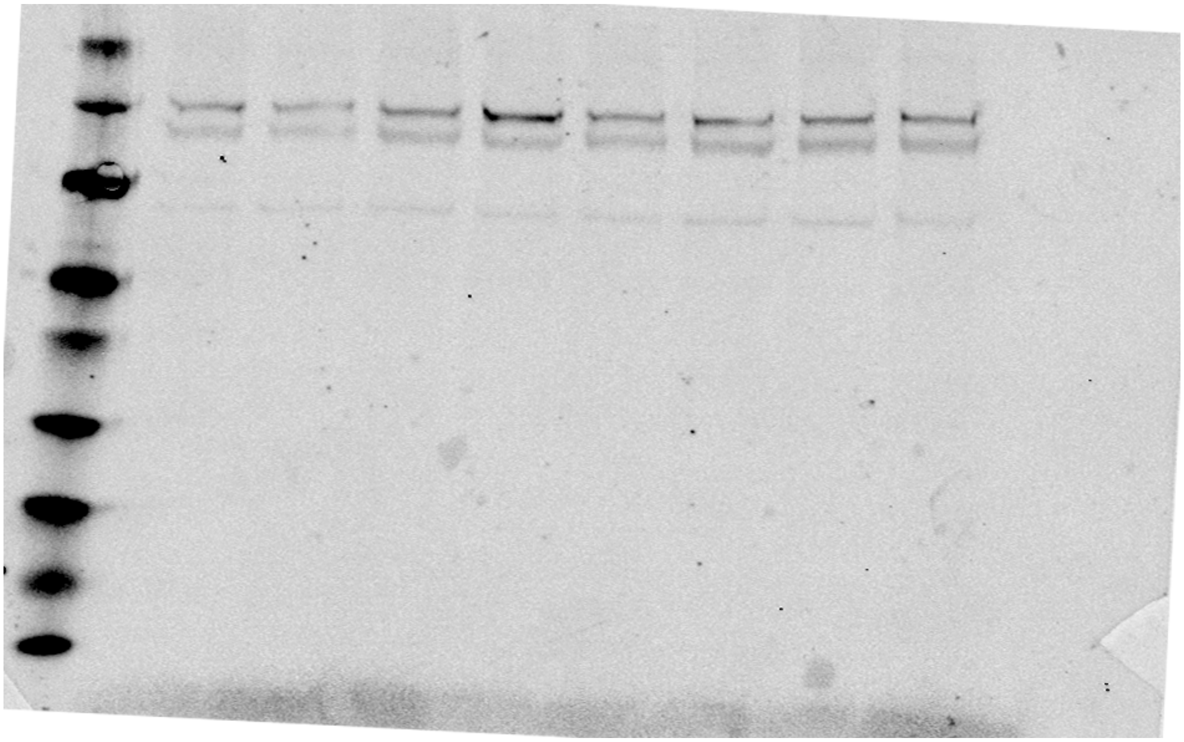

Figure 3 Blot to band key

Fig 3A S1 MBP (18.5-21.5 kDa)
- Normal, blot C, lane 5
- S-EE, blot C, lane 8
- L-EE, blot C, lane 9

Blot C

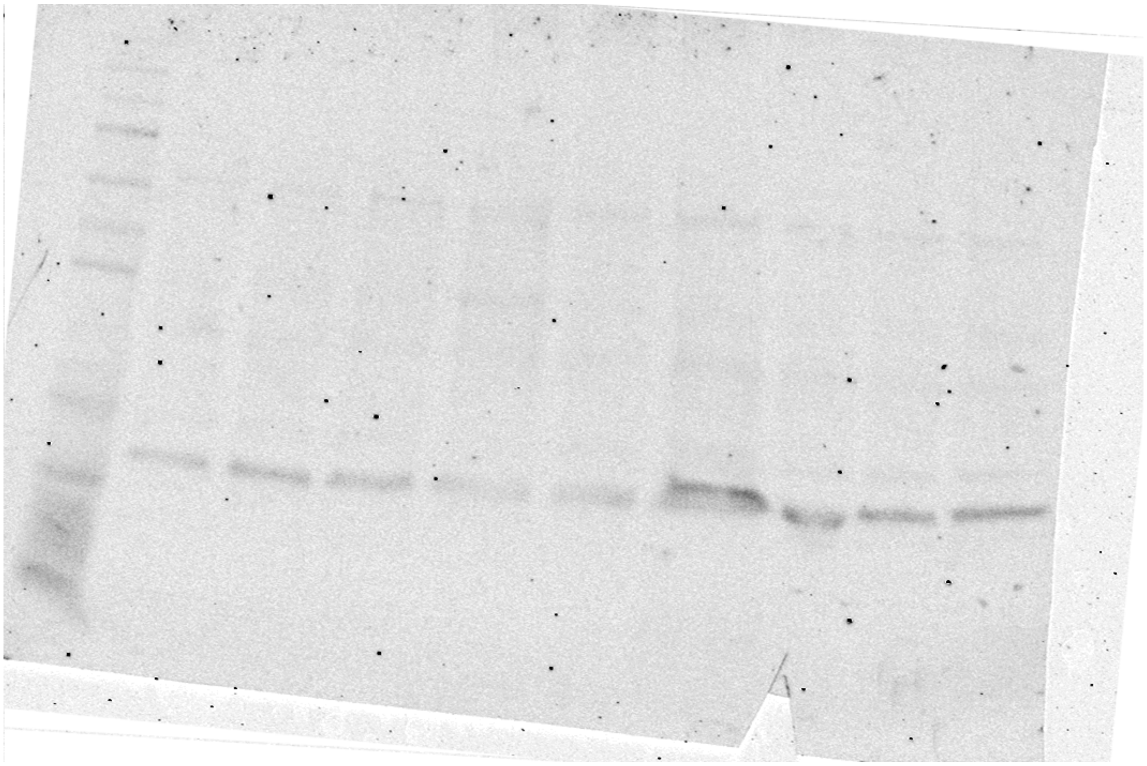

Fig 3B S1 Ube3A (110 kDa)
- Normal, blot A, lane 2
- S-EE, blot A, lane 4
- L-EE, blot A, lane 5

Blot A

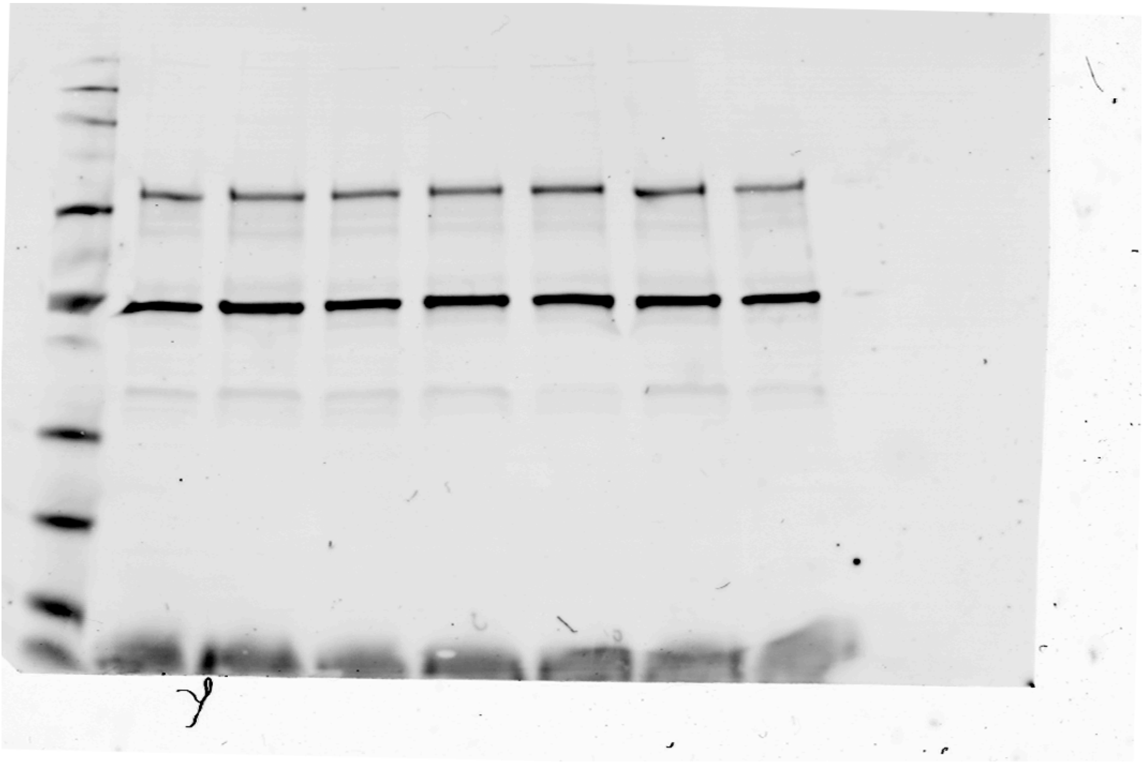

Fig 3C V1 MBP (18.5-21.5 kDa)
- Normal, blot D, lane 2
- Fix, blot A, lane 4
- MD Ipsi, blot C, lane 6
- MD Con, blot A, lane 6
- Fix.MD.Ipsi, blot C, lane 4
- Flx.MD.Con, blot E, lane 6

Fig 3C Blot A

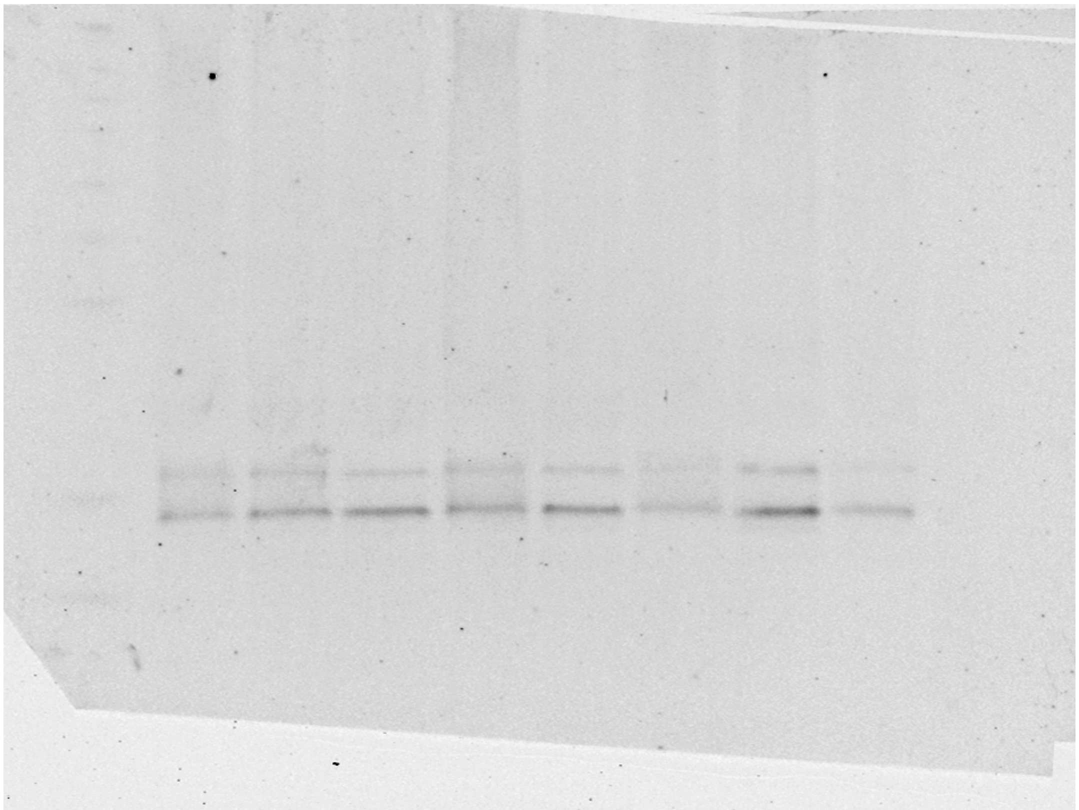

Fig 3C Blot C

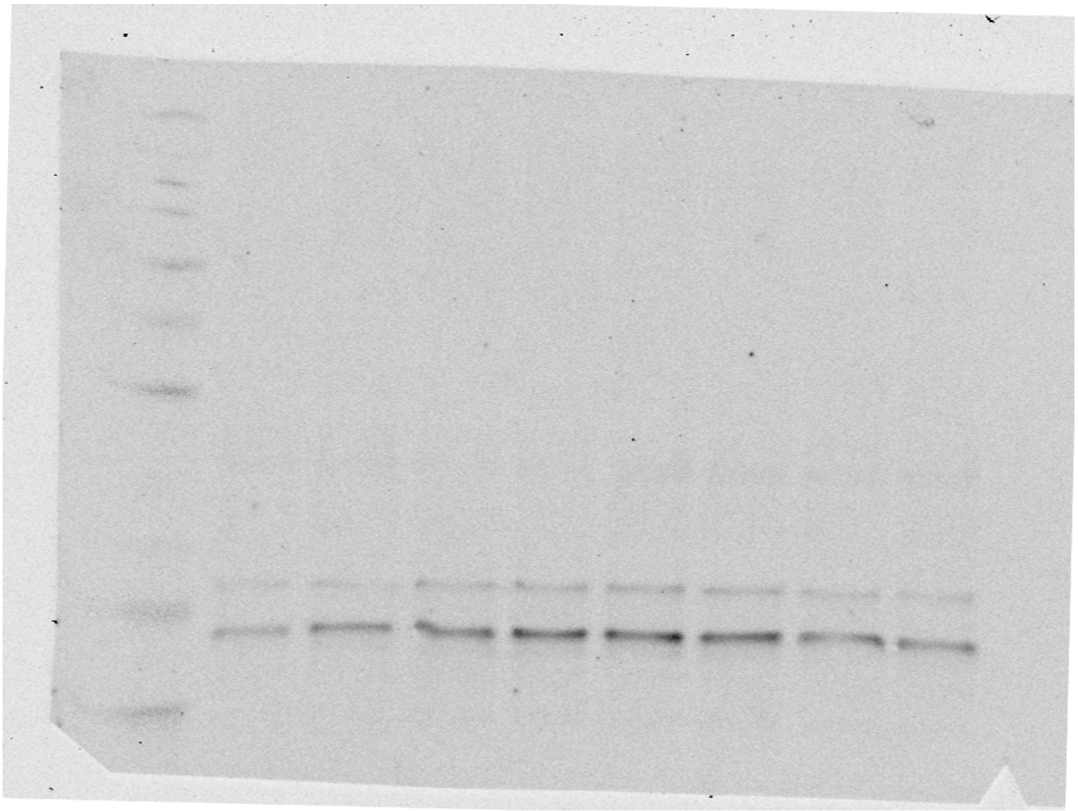

Fig 3C Blot D

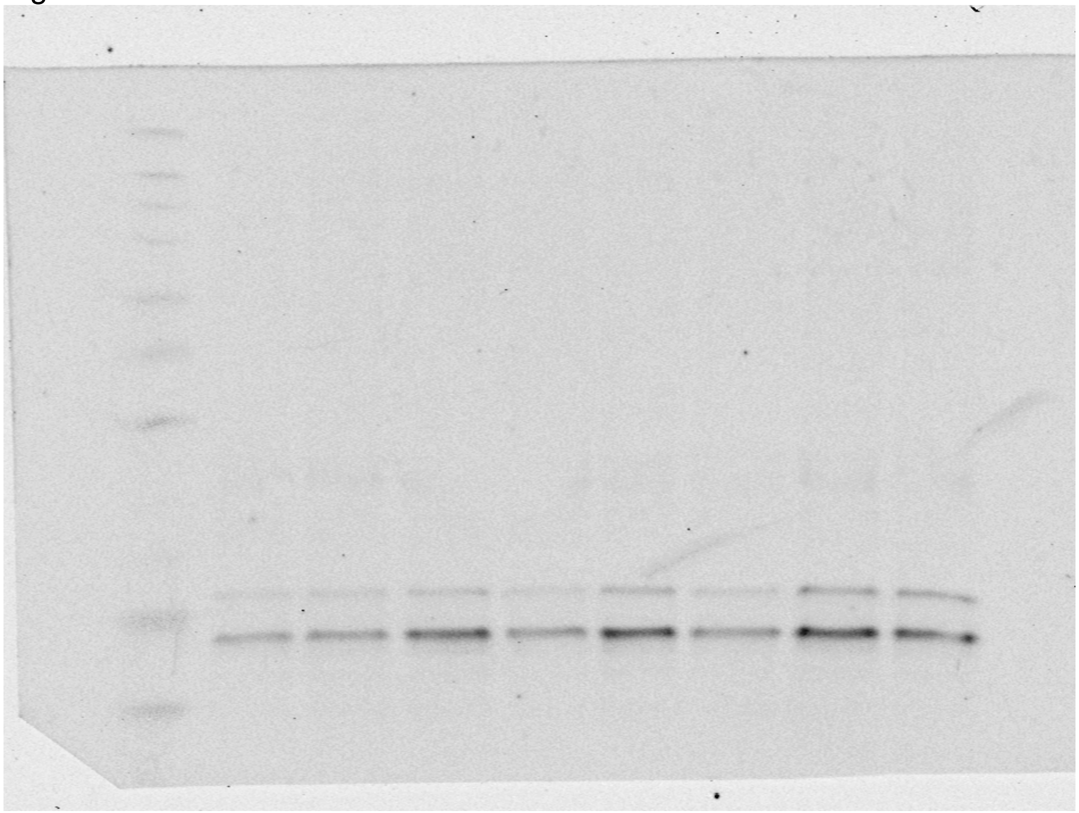

Fig 3C Blot E

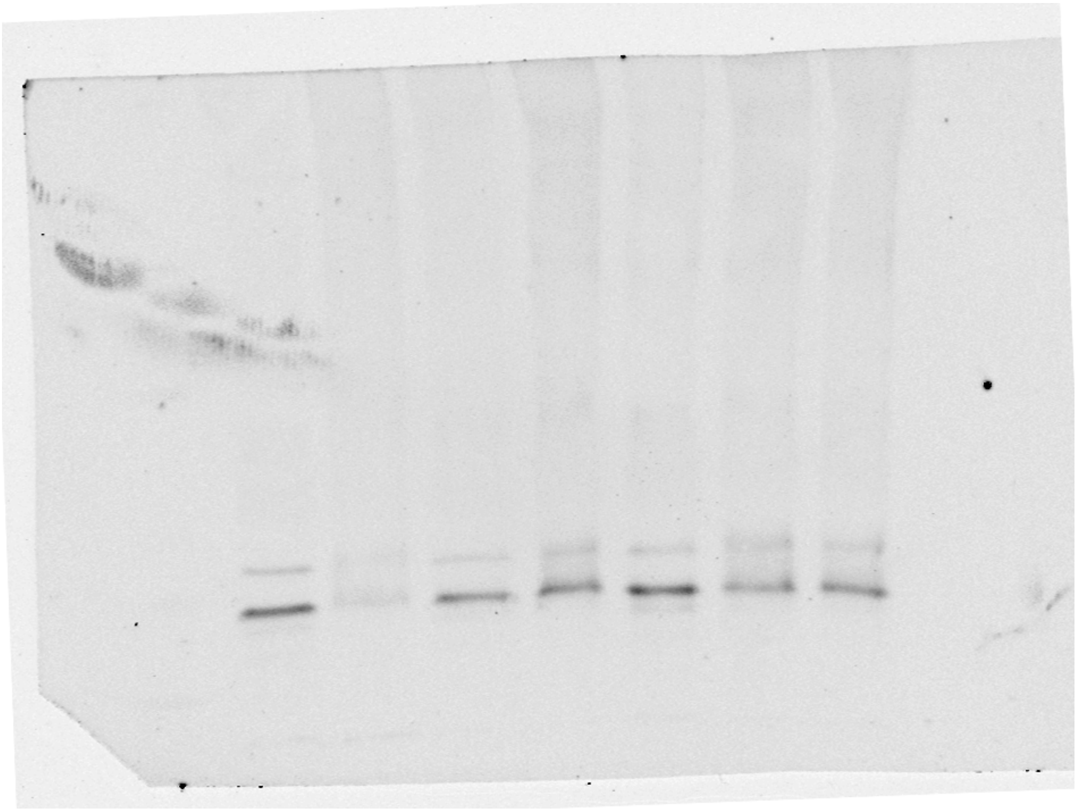

Fig 3D V1 Ube3A (110 kDa)
- Normal, blot C, lane 6
- Fix, blot A, lane 4
- MD Ipsi, blot D, lane 4
- MD Con, blot E, lane 7
- Fix.MD.Ipsi, blot C, lane 3
- Flx.MD.Con, blot B, lane 4

Fig 3D Blot A

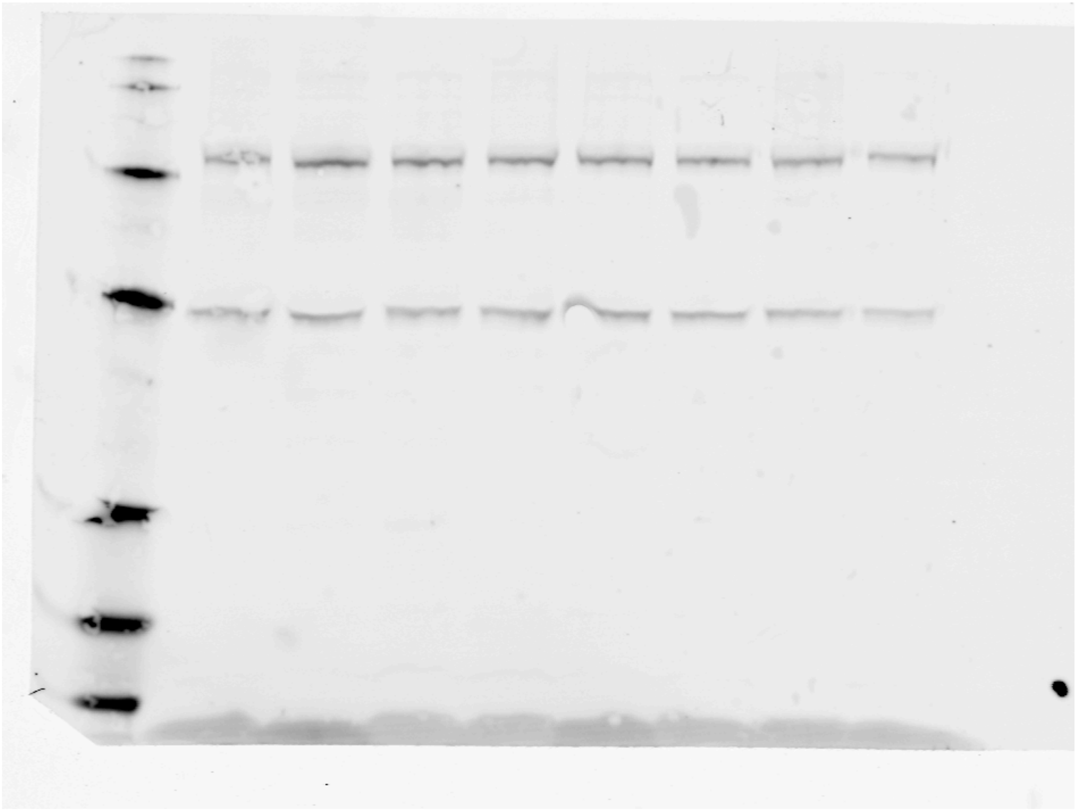

Fig 3D Blot B

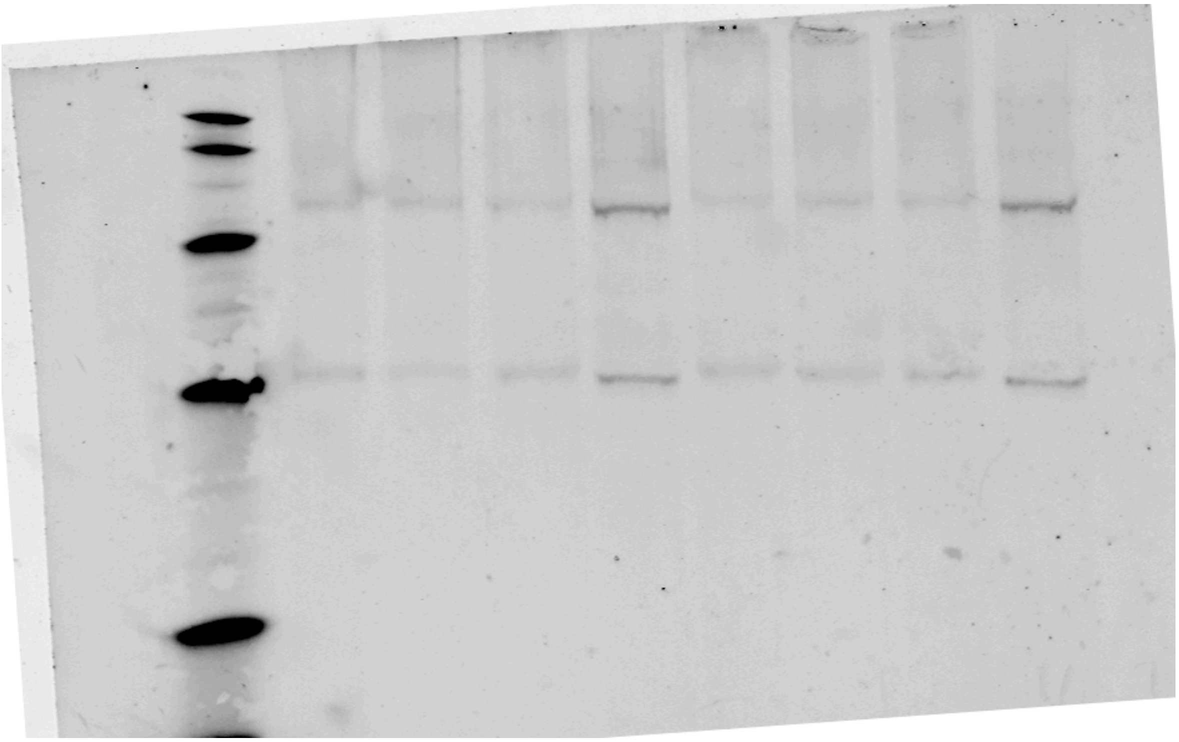

Fig 3D Blot C

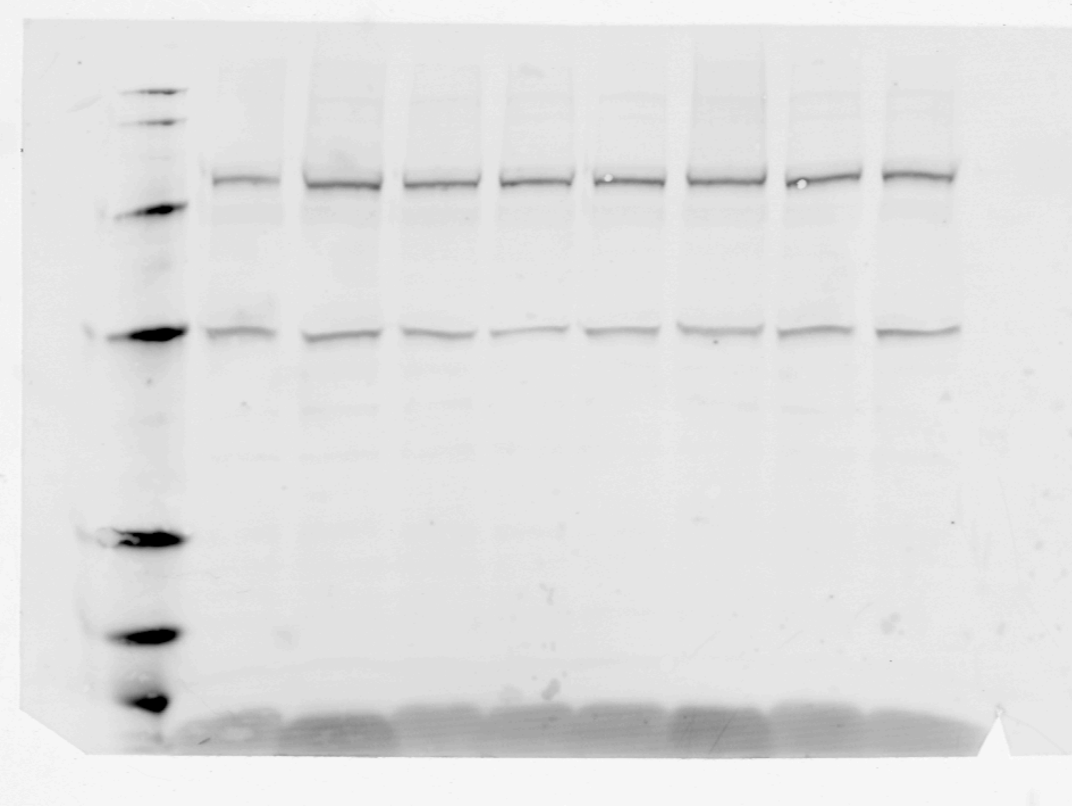

Fig 3D Blot D

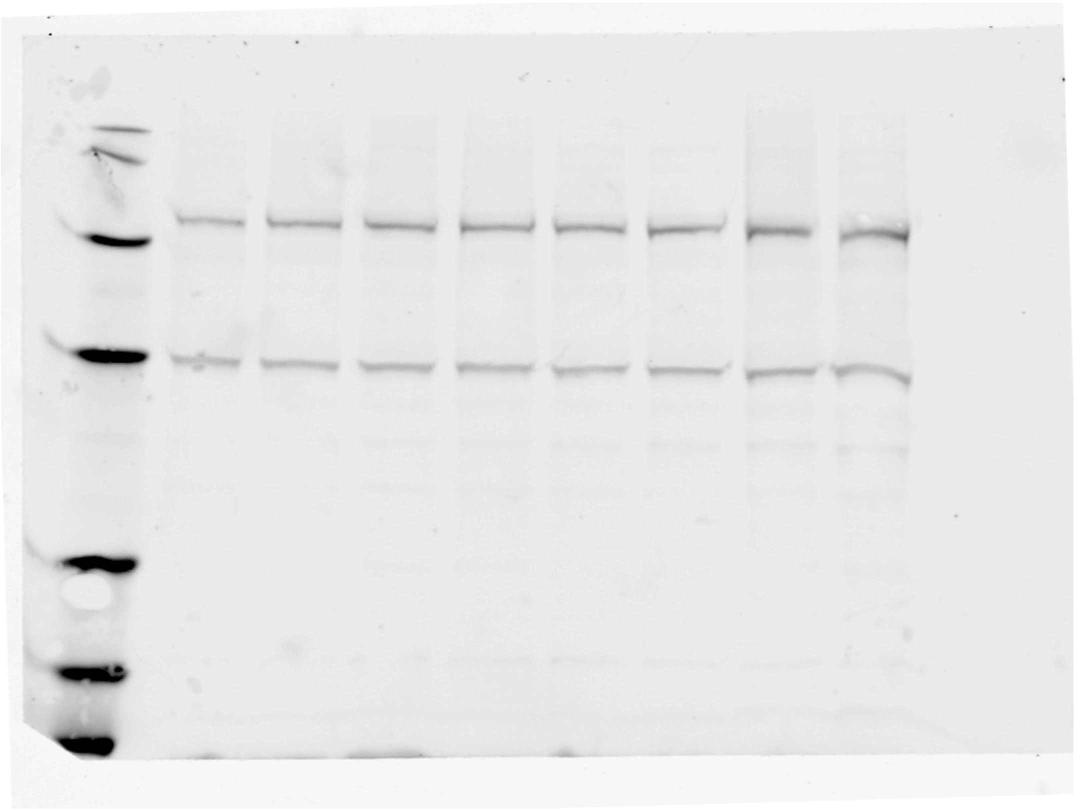

Fig 3D Blot E

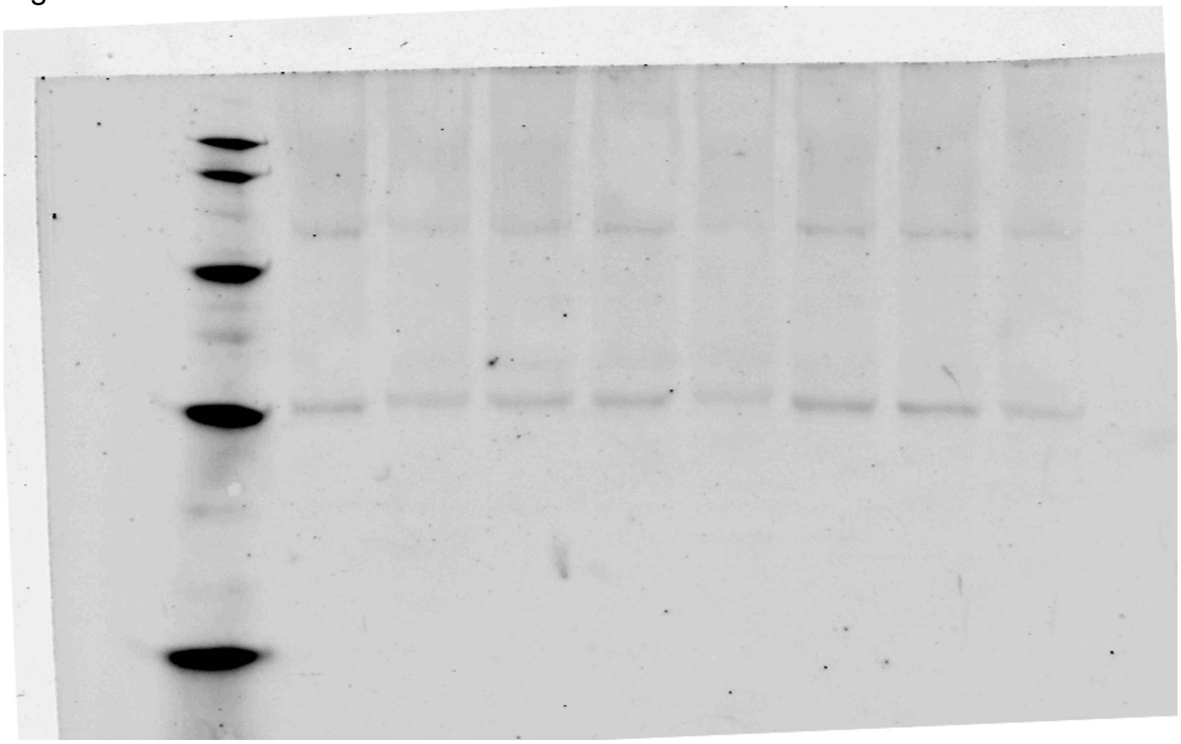

Supplement: Supplementary file 1 [file Data_Sheet_1.PDF]
