# Supplementary material for: Experience-Dependent Changes in Myelin Basic Protein Expression in Adult Visual and Somatosensory Cortex
Source: Front Cell Neurosci. 2020 Mar 17;14:56. doi: 10.3389/fncel.2020.00056 (PMC7098538; doi:10.3389/fncel.2020.00056)
Supplement: Supplementary file 2 [file Data_Sheet_2.pdf]

		Pearson's R							P-value							FDR adjusted P-threshold
		**Synapsin**	**Gephyrin**	**PSD95**	**UBE3A**	**Synaptophysin**	**GluA2**	**MBP**	**Synapsin**	**Gephyrin**	**PSD95**	**UBE3A**	**Synaptophysin**	**GluA2**	**MBP**	**(q = 0.10)**
	**Synapsin**	1.0000	0.6649	0.7753	0.8931	0.0303	0.4090	0.0536		0.1496	0.0700	0.0165	0.9545	0.4207	0.9196	
	**Gephyrin**	0.6649	1.0000	0.9268	0.9192	0.4807	0.6020	0.5357	0.1496		0.0078	0.0095	0.3345	0.2061	0.2733	
	**PSD95**	0.7753	0.9268	1.0000	0.9585	0.4323	0.3867	0.4008	0.0700	0.0078		0.0025	0.3919	0.4488	0.4310	
**Normal**	**UBE3A**	0.8931	0.9192	0.9585	1.0000	0.2655	0.5257	0.3909	0.0165	0.0095	0.0025		0.6112	0.2841	0.4435	0.0190
	**Synaptophysin**	0.0303	0.4807	0.4323	0.2655	1.0000	-0.1690	-0.0273	0.9545	0.3345	0.3919	0.6112		0.7490	0.9591	
	**GluA2**	0.4090	0.6020	0.3867	0.5257	-0.1690	1.0000	0.4166	0.4207	0.2061	0.4488	0.2841	0.7490		0.4113	
	**MBP**	0.0536	0.5357	0.4008	0.3909	-0.0273	0.4166	1.0000	0.9196	0.2733	0.4310	0.4435	0.9591	0.4113		
	**Synapsin**	1.0000	0.2227	0.2123	0.4170	0.5481	-0.7729	-0.0789		0.5960	0.6138	0.3041	0.1596	0.0245	0.8526	
	**Gephyrin**	0.2227	1.0000	0.9257	0.8068	-0.0677	-0.6222	0.5033	0.5960		0.0010	0.0155	0.8734	0.0995	0.2035	
	**PSD95**	0.2123	0.9257	1.0000	0.8636	-0.2587	-0.6408	0.3402	0.6138	0.0010		0.0057	0.5362	0.0869	0.4096	
**FLX**	**UBE3A**	0.4170	0.8068	0.8636	1.0000	0.0461	-0.5498	0.2244	0.3041	0.0155	0.0057		0.9136	0.1580	0.5932	0.0095
	**Synaptophysin**	0.5481	-0.0677	-0.2587	0.0461	1.0000	-0.2252	0.3865	0.1596	0.8734	0.5362	0.9136		0.5918	0.3443	
	**GluA2**	-0.7729	-0.6222	-0.6408	-0.5498	-0.2252	1.0000	-0.2324	0.0245	0.0995	0.0869	0.1580	0.5918		0.5797	
	**MBP**	-0.0789	0.5033	0.3402	0.2244	0.3865	-0.2324	1.0000	0.8526	0.2035	0.4096	0.5932	0.3443	0.5797		
	**Synapsin**	1.0000	0.8270	0.7657	0.9119	0.9611	-0.1982	0.5735		0.0423	0.0759	0.0113	0.0022	0.7066	0.2340	
	**Gephyrin**	0.8270	1.0000	0.8921	0.9351	0.6544	0.3060	0.5306	0.0423		0.0168	0.0062	0.1585	0.5553	0.2788	
	**PSD95**	0.7657	0.8921	1.0000	0.9015	0.6388	0.4124	0.1531	0.0759	0.0168		0.0141	0.1722	0.4165	0.7722	
**MD IPSI**	**UBE3A**	0.9119	0.9351	0.9015	1.0000	0.7782	0.2074	0.5369	0.0113	0.0062	0.0141		0.0683	0.6934	0.2721	0.0238
	**Synaptophysin**	0.9611	0.6544	0.6388	0.7782	1.0000	-0.4126	0.4596	0.0022	0.1585	0.1722	0.0683		0.4162	0.3591	
	**GluA2**	-0.1982	0.3060	0.4124	0.2074	-0.4126	1.0000	-0.2293	0.7066	0.5553	0.4165	0.6934	0.4162		0.6620	
	**MBP**	0.5735	0.5306	0.1531	0.5369	0.4596	-0.2293	1.0000	0.2340	0.2788	0.7722	0.2721	0.3591	0.6620		
	**Synapsin**	1.0000	0.6040	0.6022	0.8131	0.2276	0.3930	0.7521		0.2042	0.2059	0.0491	0.6645	0.4409	0.0846	
	**Gephyrin**	0.6040	1.0000	0.9662	0.7053	0.5108	0.7493	0.8168	0.2042		0.0017	0.1175	0.3004	0.0864	0.0473	
	**PSD95**	0.6022	0.9662	1.0000	0.5621	0.6896	0.8299	0.8552	0.2059	0.0017		0.2456	0.1296	0.0409	0.0299	
**MD CON**	**UBE3A**	0.8131	0.7053	0.5621	1.0000	-0.1050	0.2491	0.6184	0.0491	0.1175	0.2456		0.8431	0.6341	0.1907	0.0048
	**Synaptophysin**	0.2276	0.5108	0.6896	-0.1050	1.0000	0.7151	0.6627	0.6645	0.3004	0.1296	0.8431		0.1102	0.1515	
	**GluA2**	0.3930	0.7493	0.8299	0.2491	0.7151	1.0000	0.5268	0.4409	0.0864	0.0409	0.6341	0.1102		0.2829	
	**MBP**	0.7521	0.8168	0.8552	0.6184	0.6627	0.5268	1.0000	0.0846	0.0473	0.0299	0.1907	0.1515	0.2829		
	**Synapsin**	1.0000	0.1563	-0.1698	-0.1266	-0.3116	-0.0409	-0.0128		0.7117	0.6876	0.7652	0.4525	0.9235	0.9760	
	**Gephyrin**	0.1563	1.0000	0.8097	0.3895	0.3005	0.1642	0.1596	0.7117		0.0149	0.3402	0.4696	0.6977	0.7058	
	**PSD95**	-0.1698	0.8097	1.0000	0.2245	0.5416	0.3970	0.5560	0.6876	0.0149		0.5930	0.1656	0.3302	0.1524	
**FLX MD IPSI**	**UBE3A**	-0.1266	0.3895	0.2245	1.0000	0.7149	-0.2191	-0.1147	0.7652	0.3402	0.5930		0.0463	0.6022	0.7869	0.0048
	**Synaptophysin**	-0.3116	0.3005	0.5416	0.7149	1.0000	-0.1070	0.3571	0.4525	0.4696	0.1656	0.0463		0.8010	0.3852	
	**GluA2**	-0.0409	0.1642	0.3970	-0.2191	-0.1070	1.0000	0.5548	0.9235	0.6977	0.3302	0.6022	0.8010		0.1535	
	**MBP**	-0.0128	0.1596	0.5560	-0.1147	0.3571	0.5548	1.0000	0.9760	0.7058	0.1524	0.7869	0.3852	0.1535		
	**Synapsin**	1.0000	0.5401	0.4506	0.4483	0.2184	-0.0823	0.1759		0.1670	0.2625	0.2652	0.6033	0.8464	0.6769	
	**Gephyrin**	0.5401	1.0000	0.9892	0.9889	0.8194	-0.1984	0.3298	0.1670		0.0000	0.0000	0.0128	0.6377	0.4250	
	**PSD95**	0.4506	0.9892	1.0000	0.9906	0.8335	-0.1601	0.3816	0.2625	0.0000		0.0000	0.0101	0.7050	0.3509	
**FLX MD CON**	**UBE3A**	0.4483	0.9889	0.9906	1.0000	0.8454	-0.1687	0.3343	0.2652	0.0000	0.0000		0.0082	0.6896	0.4183	0.0286
	**Synaptophysin**	0.2184	0.8194	0.8335	0.8454	1.0000	-0.3026	0.3148	0.6033	0.0128	0.0101	0.0082		0.4664	0.4477	
	**GluA2**	-0.0823	-0.1984	-0.1601	-0.1687	-0.3026	1.0000	-0.0179	0.8464	0.6377	0.7050	0.6896	0.4664		0.9665	
	**MBP**	0.1759	0.3298	0.3816	0.3343	0.3148	-0.0179	1.0000	0.6769	0.4250	0.3509	0.4183	0.4477	0.9665		

Supplementary Datasheet S2. A Table of Pearson's R values corresponding to the correlation matrices presented in Figure 4. The correlations between pairs of proteins for each of the treatment conditions presented in Figure 4. The corresponding p-values for the correlations are shown, and p-values below the False Discovery Rate (q = 0.10) are coloured red.
